# Antihyperlipidemic Effect of Flavonoids and Saponins from *Pyracantha fortuneana* Fruits on L02 Cells and *Caenorhabditis elegans*

**DOI:** 10.3390/foods14203499

**Published:** 2025-10-14

**Authors:** Yunfang Hao, Yinhong Wang, Kexin Hao, Yimeng Li, Longmei Geng, Liang Zhu, Jianguo Jiang

**Affiliations:** 1 Jiangmen Key Laboratory of Traditional Chinese Medicine Ingredients and Their Mechanisms of Action, Guangdong Jiangmen Chinese Medicine College, Jiangmen 529000, China; 2 School of Food Science and Engineering, South China University of Technology, Guangzhou 510640, Chinazhuliang@scut.edu.cn (L.Z.); 3 Dermatology Hospital, Southern Medical University, Guangzhou 510091, China

**Keywords:** *Pyracantha fortuneana*, chemical composition, antihyperlipidemic activities, L02 cells, *Caenorhabditis elegans*

## Abstract

In China, *Pyracantha fortuneana* has been consumed as a nutritious plant to improve indigestion. In the current study, the main chemical composition of *P. fortuneana* fruits was extracted and analysed for composition. Free fatty acids (FFA)-induced normal human hepatic L02 cells were used to construct a high-fat cell model, and lipid deposition in *Caenorhabditis elegans* was induced by a high concentration of glucose to study the anti-hyperlipidemic effects of the main components. The results showed that the flavonoid content of PFF (*P. fortuneana* Flavonoid Fractions) was 80.28%, and it contained various flavonoids such as epicatechin, isoquercetin, rutin, quercetin, and myricitrin, while the saponin content of PFS (*P. fortuneana* Saponin Fractions) was 74.4%, and it contained saponins such as shionone, crategolic acid, and ursolic acid. PFF and PFS significantly reduced the content of lipid droplets in high-fat L02 cells, inhibited mitochondrial membrane potential decline, regulated the fat accumulation by up-regulating the relative mRNA expression levels in the Nrf2/ARE signaling pathway, as well as the CPT-1 and SIRT1 genes in lipid metabolism. Meanwhile, both PFF and PFS significantly reduced lipid deposition, reactive oxygen species (ROS) levels, malondialdehyde (MDA) content, and catalase activity in *C. elegans*. In summary, our results indicated that the flavonoids and saponins of *P*. *fortuneana* are potential natural products in antihyperlipidemic effect.

## 1. Introduction

With the shift in modern dietary patterns, dyslipidemia induced by excessive dietary lipid intake has become an increasingly prominent public health issue, which is closely associated with an elevated risk of chronic diseases such as atherosclerosis and non-alcoholic fatty liver disease [1]. Abnormal elevation of blood lipid indicators, including low-density lipoprotein cholesterol (LDL-C), total cholesterol (TC), and triglyceride (TG), serves not only as a key basis for assessing cardiometabolic risk, but also as an important target for screening dietary components with lipid-modulating functions [2,3].

Currently, the primary drugs used to treat hyperlipidemia are statins, such as atorvastatin and simvastatin, which regulate lipid metabolism and cholesterol synthesis [4]. However, they can have specific side effects, including impairment of liver and kidney function. Furthermore, the main treatment drugs for hyperlipidemia also include probucol and ezetimibe [5]. Although these therapeutic drugs have good effects, the side effects should not be ignored. In recent years, natural herbal resources have been increasingly valued for their species richness and wide range of biological activities [6]. Against this backdrop, the exploration of functional components derived from edible plant resources that can help regulate blood lipids has become a research focus in the fields of food science and preventive medicine [7]. These natural products, sourced from medicinal and edible plants, generally exhibit favorable biocompatibility and long-term safety for consumption, offering new perspectives for managing suboptimal health conditions through dietary intervention.

*Pyracantha fortuneana* is a wild, evergreen shrub belonging to the genus *Pyracantha*, family Rosaceae, which is widely distributed in eastern Asia and southern Europe. The primary edible part of *P. fortuneana* is its fruit, although its leaves and roots also contain bioactive compounds [8]. Nevertheless, the fruit serves as the primary source of both nutritional and medicinal value, with traditional functions including promoting fluid production, quenching thirst, and aiding digestion [9]. Ripe fruits can be consumed directly raw, offering a soft, sweet, and mildly fragrant flavor, which can also be processed into jam, juice, or fruit wine [8]. *P. fortuneana* is generally considered non-toxic, a perception supported by its long history of dietary use. However, excessive intake should be avoided, as the high content of dietary fiber and organic acids may cause gastrointestinal irritation, potentially leading to abdominal distension, diarrhea, or other digestive discomforts [9]. Recent studies have shown that *P. fortuneana* possesses significant medicinal and edible value, and the extraction of *P. fortuneana* has demonstrated a variety of biological activities, including antioxidant [10], antitumor [8], hepatoprotective [11], hypoglycemic [12], and hypolipidemic [9] effects. Previous studies have demonstrated that extracts from *P. fortuneana* have a strong radical scavenging ability and marked α-glucosidase inhibition activity (IC 50 value: 0.37 mg/mL) [12]. Additionally, some *P. fortuneana* extracts have been shown to improve endogenous antioxidant activity and ameliorate hyperlipidemia and obesity in rodents [13].

This study aimed to extract and isolate flavonoids and saponins with anti-hyperlipidemic activity from the edible fruit of *P. fortuneana*, and to further investigate the functions and potential mechanisms of these natural dietary components in modulating lipid metabolism by establishing a high-fat L02 cell model and a high-glucose *C. elegans* model. The results may provide a reference for the development of *P. fortuneana* as a potential functional food or natural dietary supplement with lipid-lowering properties.

## 2. Materials and Methods

### 2.1. Materials and Reagents

The dried *P. fortuneana* fruits were purchased from Zhuoran Flower Tea Trading Co., Ltd., Hangzhou, China and preserved at the School of Food Science and Engineering, South China University of Technology, with the serial number 202209001. L02 Human hepatic cells were purchased from ATCC cell bank. *C. elegans* Bristol N2 and *Escherichia coli* OP50 were purchased from American Caenorhabditis Genetics Center. L02 human hepatic cells, *C. elegans* Bristol N2 and *Escherichia coli* OP50 were preserved at the School of Food Science and Engineering, South China University of Technology. Rutin and Ginsenoside Rb1 standards were purchased from Shanghai yuanye Bio-Technology Co., Ltd. (Shanghai, China) Dulbecco’s Modified Eagle Medium (DMEM), fetal bovine serum (FBS), phosphate-buffered saline (PBS, pH 7.4), 0.25% trypsin and penicillin–streptomycin were provided by Gibco Life Technologies (Grand Island, NY, USA). Oleic acid (OA), palmitic acid (PA) and Oil Red O were purchased from Guangzhou Xincan Co., Ltd., Guangzhou, China. Glucose was purchased from Macklin Biochemical Technology Co., Ltd., Shanghai, China. Triglyceride (TG), total cholesterol (TCHO), low-density lipoprotein cholesterol (LDL-C), Superoxide dismutase (SOD) assay kit, glutathione peroxidase (GSH-Px) assay kit, malondialdehyde (MDA) assay kit, Catalase Assay Kit (CAT) and mitochondrial membrane potential assay kit (JC-1) were purchased from Beyotime Biotechnology, Shanghai, China. Reactive oxygen species (ROS) was purchased from Dalian Meilun Biotechnology Co., Ltd., Dalian, China. EZ-press RNA Purification Kit, Color Reverse Transcription Kit and 2 × Color SYBR Green qPCR Master Mix (ROX2plus) were obtained from EZBioscience.

### 2.2. Extraction and Isolation of the Extracts from P. fortuneana Fruits

Commercially procured dried *P. fortuneana* fruits were mechanically pulverized using a high-speed grinder, and the resulting powder was sieved through a 40-mesh screen. The extraction parameters for flavonoids and saponins, including ethanol concentration, liquid-to-solid ratio, temperature, and duration, were determined based on pre-experimental optimization conducted by our research group, with reference to established methods for extracting similar bioactive compounds from Rosaceae fruits reported in the literature [14,15,16]. The optimized extraction conditions for total flavonoids involved the use of 55% ethanol, a liquid-to-solid ratio of 20 mL/g, and a temperature of 90 °C, while total saponins were extracted with 65% ethanol, a liquid-to-solid ratio of 18 mL/g, and a temperature of 85 °C. Each sample underwent two extraction cycles, each lasting 2.5 h. The resulting crude extracts were concentrated under reduced pressure using a rotary evaporator to recover ethanol and remove the majority of the solvent, yielding concentrated extracts that were subsequently dried at 60 °C. The dried material was redissolved in distilled water to a concentration of 5 mg/mL and subjected to separation on a D140 macroporous resin column, with sequential elution using distilled water, 30% ethanol, 50% ethanol, and 70% ethanol solutions. The collected eluates were concentrated again using a rotary evaporator and dried at 60 °C. The flavonoid and saponin contents of each fraction were quantitatively analyzed, and the fraction exhibiting the highest content of each target compound was collected and designated as PFF for flavonoids and PFS for saponins, respectively.

### 2.3. Determination of Flavonoid and Saponin Contents

The flavonoid content was determined by NaNO_2_-Al(NO_3_)_3_-NaOH method [17]. 2 mg of rutin was dissolved in 10 mL of 30% ethanol to obtain 0.2 mg/mL of rutin standard solution. Subsequently, the reaction was carried out with 5% NaNO_2_ solution for 6 min and then 10% Al (NO_3_)_3_ solution was added and the reaction was continued for 6 min. Finally, 1 mol/L NaOH solution was added for 15 min and then measured at 510 nm. The standard curve was plotted with the concentration of rutin as the horizontal coordinate and the absorbance value as the vertical coordinate, and the regression curve equation measured in this experiment was y = 12.906x + 0.0119, R^2^ = 0.9973.

The saponin content was determined by vanillin–glacial acetic acid–perchloric acid method [18]. 6 mg of ginsenoside Rb1 standard was taken and dissolved in 10 mL of 30% ethanol solution to obtain 0.6 mg/mL of ginsenoside Rb1 standard solution. Sequentially, 0, 0.1, 0.2, 0.3, 0.4, 0.5, 0.6 mL of the ginsenoside Rb1 standard solution was placed in a clean test tube and dried in an oven. 0.2 mL of 5% vanillin–glacial acetic acid solution and 0.8 mL of perchloric acid were added into each test tube, and the samples were put at 60 °C for 20 min in a thermostatic water bath, and then after 3 min of ice bath, 5 mL of ice acetic acid solution was added, and the results were determined at the wavelength of 540 nm. The standard curve was plotted with the concentration of ginsenoside Rb1 as the horizontal coordinate and the absorbance value as the vertical coordinate, and the regression curve equation measured was y = 2.7771x − 0.0177, R^2^ = 0.9982.

### 2.4. LC-MS/MS Analysis

The main ingredients in PFF and PFS were determined by liquid chromatography (Thermo Scientific™ UltiMate™ 3000 BioRS system) in tandem with Thermo ScientificTM Q Exactive Plus mass spectrometer (Thermo Fisher Scientific Inc., Bermen, Germany). The 50 ppm solutions of PFF and PFS were prepared using 50% acetonitrile as solvent. The chromatographic separation was performed on an ACQUITYUPLC HSS T3 column (2.1 mm × 100 mm, 1.8 μm) of which the mobile phase consisted with 0.1% formic acid (A) and acetonitrile (B). The injection volume was 5 μL and the flow rate was 0.2 mL/min. The sample conditions were as follows: 0–18 min, 2–50% B; 18–21 min, 50–95% B; 21–23 min, 95% B; 23–25 min, 95–2% B. The mass spectrometry conditions were as follows: the ion source was an electrospray ionization source (ESI); detection was performed using the positive ion mode and negative ion mode. The samples were scanned by primary mass spectrometry (MS) and secondary mass spectrometry (MS2). The primary mass spectrometry was performed with a full scan (Full MS) with a resolution of 70,000 and a scanning range of 70–1050 *m*/*z*, while the secondary mass spectrometry was performed with a data-dependent secondary scan (dd-MS2) with a resolution of 17,500.

### 2.5. Protective Effects of PFF and PFS on High-Fat L02 Cells

#### 2.5.1. Cell Culture

L02 Human hepatic cells were cultured in DMEM complete medium (10% fetal bovine serum + 1% penicillin-streptomycin + 90% DMEM basic medium) and were incubated at 37 °C in a humidified atmosphere containing 5% CO_2_. All cells used for the study were passaged 5–15 times.

#### 2.5.2. Screening of Safe Concentration of PFF and PFS

The MTT assay was used to determine the cell viability of L02 cells [19]. In brief, the L02 cells were seeded at a density of 5 × 10^3^ cells/well in 96-well plates. After incubating for 24 h, the cells were treated with various samples for another 24 h. Subsequently, 10 μL of 5 mg/mL MTT solution was added into each well to incubate for 4 h, then the medium was removed and 150 μL DMSO was added. Finally, the cell viability was determined at 490 nm to detect the impact of each sample on the survival rate of L02 cells.

#### 2.5.3. Preparation and Establishment of Safe Concentrations of FFA

The preparation of FFA solution was referred to the previous report [20]. Palmitic acid (PA) and oleic acid (OA) were dissolved in 0.1 M NaOH solution at 70 °C to produce 100 mM PA and OA solutions, respectively. The 100 mM PA solution was mixed with 100 mM OA solution in the ratio of 1:2 by volume. The mixture was then diluted with an equal volume of 10% bovine serum albumin (BSA) to produce a 50 mM stock solution of FFA.

L02 cells were inoculated into a 96 well plate according to the cell density of 5 × 10^3^ cells per well and cultured for 24 h. Then, the culture medium was aspirated and washed with PBS, and the FFA stock solution was diluted to 100, 200, 300, 400 and 500 µmol/L with complete medium and added into 96-well plates, three parallels were set up in each group and the viability was determined by MTT method after 24 h of incubation. The concentration of FFA that is non-toxic to L02 cells was selected for modelling.

#### 2.5.4. Oil Red O Staining

L02 cells were seeded into 24-well plates with complete medium at a density of 1 × 10^5^ cells/mL, 500 µL per well, and incubated in a cell culture incubator for 24 h. After aspirating the medium, complete medium was added to the control group; concurrently, the model and sample groups received 300 µmol of FFA. Following a 24 h incubation period, complete medium was added to both the control and model groups. The sample groups, however, were treated with medium containing different concentrations of PFF and PFS (100, 200, and 400 µg/mL). After an additional 24 h of incubation, the medium was fixed with 4% paraformaldehyde for 30 min. The cells were then stained for 0.5 h by adding 0.5% oil red O staining solution under light-avoidance conditions, and the cells were observed under a microscope (Olympus Corporation, Tokyo, Japan). In each step, L02 cells were washed three times with PBS.

#### 2.5.5. Determination of TCHO, TG, LDL, MDA, SOD and GSH Levels

The L02 cells were seeded into 6-well plates at 1 × 10^5^ cells per well for 24 h. After incubating in 1.5 mL medium containing 300 µmol of FFA for 24 h, 100–400 µg/mL of PFF and PFS were added, and the cells were then incubated for an additional 24 h. The cells were subsequently collected and broken by sonication at 4 °C for 90 s. Then the cell lysate was centrifuged and the supernatant was collected, and the determination of TCHO, TG, LDL, MDA, SOD and GSH contents were determined according to the kit instructions, and the value was corrected by the cell protein content.

#### 2.5.6. Determination of Mitochondrial Membrane Potential

The L02 cells were collected after administration and were processed according to the instructions of the assay kit (JC-1). The cellular mitochondrial membrane potential was monitored by flow cytometry.

#### 2.5.7. qRT-PCR Analysis

RNA extraction, reverse transcription and amplification reactions were performed according to the kit instructions to detect the effects of PFF and PFS on the expression of genes related to lipid metabolism in L02 cells. The primer sequences used for the relevant genes are shown in Table 1. The purity of total RNA was determined by Nanodrop 2000 (Thermo Fisher Scientific, Shanghai, China). Detection was performed using an ABI QuantStudio7 Real Time (qRT-PCR) system instrument (Thermo Fisher Scientific, Waltham, MA, USA) with β-actin as an endogenous control.

### 2.6. Effects of PFF and PFS on Lipid Deposition in C. elegans

#### 2.6.1. Cultivation of *C. elegans*

Wild-type N2 Bristol *C. elegans* were obtained from the Caenorhabditis Genetics Center. All *C. elegans* were cultured on NGM solid medium and fed with *Escherichia coli* OP50 at a temperature of 20 °C. After contemporaneous incubation and transfer to plates containing *E. coli* OP50 and continued incubation for 23 h, worms entered the L1 stage. After continuing to incubate at 20 °C for 43 h, the worms entered the L4 stage.

#### 2.6.2. The Toxicity of PFF and PFS to *C. elegans*

The sample solutions of different concentrations were mixed with the inactivated bacterial suspension in equal volumes to obtain the sample *E. coli* mixtures with concentrations of 50, 100, 200, 400 and 800 μg/mL, and 100 μL of the mixtures were coated in NGM plates containing 25 mg/L pentafluorouracil. The same number of L4-stage worms were placed in the plates coated with different concentrations of the samples, and the survival and death of worms were recorded after 48 h of incubation at 20 °C.

#### 2.6.3. Establishment of Fat Accumulation Model

The inactivated suspension was spread on NGM plates containing different concentrations of glucose (5, 10, 15 mmol/L) and 25 mg/L pentafluorouracil, and the same number of L4-stage worms were placed in the plates and incubated at 20 °C for 24 h. Worms were collected, freeze-thawed in liquid nitrogen three times, and then stained with Oil Red O staining solution.

#### 2.6.4. Effects of PFF and PFS on Lipid Accumulation in *C. elegans*

Equal numbers of L4-stage worms were cultured in NGM plates containing 15 mmol/L glucose for 24 h, and then transferred to plates containing 200, 400, and 800 μg/mL of PFF and PFS for another 24 h. Worms that had been repeatedly freeze-thawed in liquid nitrogen three times were stained with Oil Red O staining solution.

#### 2.6.5. Determination of TCHO, TG, LDL, MDA, CAT and ROS Levels

Worms from each group were collected and washed three times with M9 solution. The worms were homogenized and the TCHO, TG, LDL, MDA and CAT levels were determined according to the kit instructions. In addition, worms from each group were collected, treated with DCFH-DA and photographed using an inverted fluorescence microscope to detect ROS levels.

### 2.7. Statistical Analysis

All experimental data were expressed as mean ± S.D. Statistical analyses were performed by SPSS Statistical 26 Software using one-way ANOVA followed by Duncan test. Differences with *p* < 0.05 were considered statistically significant, *p* < 0.01 was considered highly statistically significant. The drawing was done using Origin 2023.

## 3. Results and Discussion

### 3.1. Main Components of PFF and PFS

The fraction with the highest flavonoid content was named PFF based on the standard curve of flavonoids and its flavonoid content was 80.28%. The fraction with the highest saponin content was named PFS based on the standard curve for saponins, and its saponin content was 74.4%.

**Figure 1:**
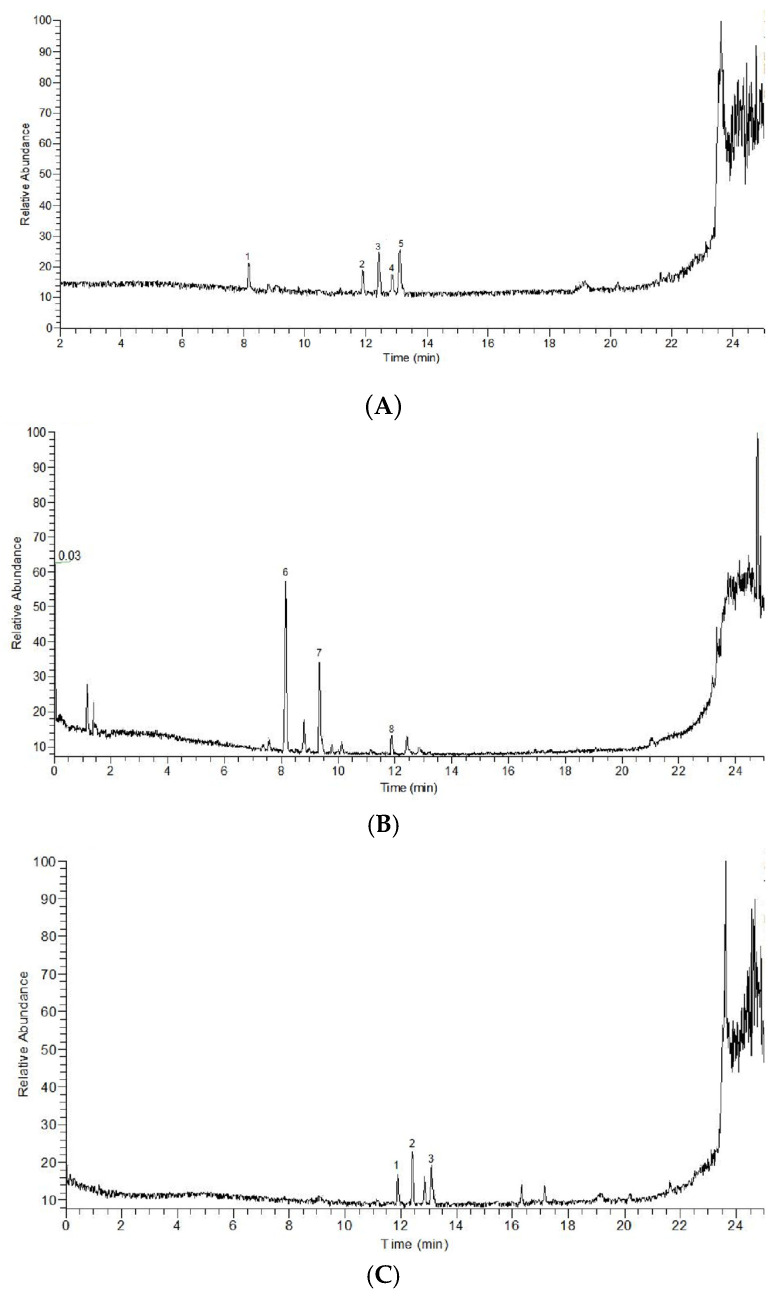

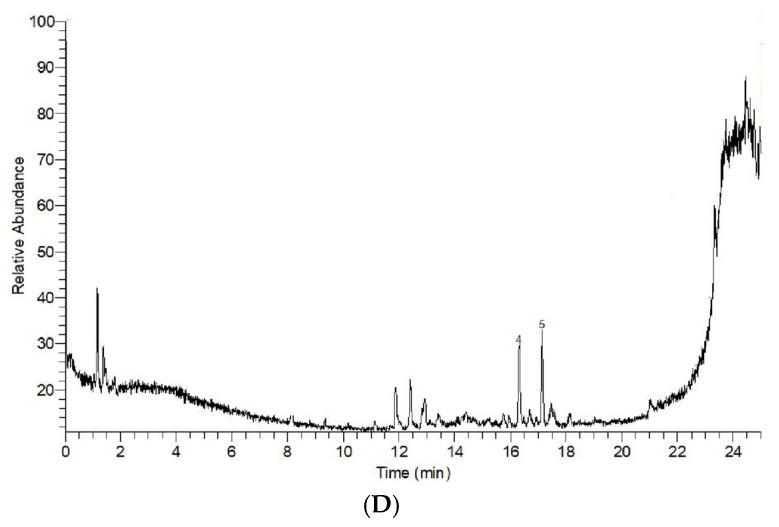
Positive ion flow diagram (**A**), negative ion flow diagram (**B**) of PFF; positive ion flow diagram (**C**) and negative ion flow diagram (**D**) of PFS. 1–5 in (**A**) represent Epicachin, Isoquercetin, Rutin, Hydroxysafflor yellow A and 5,7,4′Trihydroxy-6-iso Pentenyl isoflavone, respectively. 6–8 in (**B**) represent Quercetin, Baicalein and Myricitrin, respectively. 1–3 in (**C**) represent Shionone, Crategolic acid and Ursolic Acid, respectively. 4 and 5 in (**C**) represent Cocoa butter acid F and (15z)-9,12,13-Trihydroxy-15-octadecenoic acid.

The total ion flow diagrams of PFF and PFS were shown in Figure 1A, Figure 1B, Figure 1C and Figure 1D, respectively. It could be seen that there was a big difference in the peak time and peak area between them. The main components of PFF and PFS were shown in Table 2.

The primary flavonoid constituents in PFF comprise epicatechin, isoquercetin, rutin, hydroxysafflor yellow A, quercetin, baicalein, myricitrin, and 5,7,4′Trihydroxy-6-iso Pentenyl isoflavone. These compounds exert synergistic effects by targeting the modulation of key pathways that regulate glucose and lipid metabolism. Epicatechin attenuated high-fat-induced pancreatic β-cell apoptosis, oxidative and endoplasmic reticulum stress, and has been shown to have a favorable therapeutic effect on diabetes mellitus as well as cancer [21]. Isoquercetin promotes bile acid biosynthesis via alternative pathway activation and intestinal FXR-FGF15 signaling inhibition, thereby reducing hepatic TC and TG accumulation in high-fat diet-fed mice [22]. Rutin modulates lipid metabolism through AMPK pathway-mediated downregulation of lipogenic proteins (FASN, SREBP1, SCD1) [23], while simultaneously mitigating oxidative stress and inflammation via Nrf2/ARE system activation [24]. Hydroxysafflor yellow A ameliorates atherosclerotic lesions by suppressing PI3K/AKT/mTOR-NF-κB signaling to reduce vascular endothelial growth factor C expression and secretion [25], and alleviates alcohol-induced liver injury through the PI3K/Akt and STAT3/NF-κB pathways modulation, conferring integrated antioxidative, anti-inflammatory, and anti-apoptotic effects [26]. Quercetin mitigates glucolipid metabolic dysregulation by ameliorating insulin resistance, modulating enzymatic activity, inhibiting lipogenesis, modulating gut microbiota, inducing white adipocyte browning, and attenuating oxidative stress and inflammation [27]. Baicalein enhances hepatic and skeletal muscle insulin sensitivity via glucagon-like peptide-1 receptor-dependent PI3K/AKT signaling, and facilitates skeletal muscle glucose uptake through the Ca^2+^/CaMKII-AMPK-GLUT4 signal pathway [28]. Myricitrin promotes white adipose browning by upregulating AMPK/SIRT1-dependent browning markers, including UCP1 and PRDM16, while concurrently reducing lipogenesis and pro-inflammatory cytokine production [29,30].

The primary saponin constituents in PFF include shionone, crategolic acid, ursolic acid, cocoa butter acid F, and (15z)-9,12,13-trihydroxy-15-octadecenoic acid, with its lipid-lowering activity being closely associated with multi-target regulatory mechanisms. Shionone exerts anti-inflammatory effects by modulating inflammatory targets, including TNF-α, STAT3, NLRP3, and NF-κB [31]. Crategolic acid activates the AMPK/mTOR/ATG1 pathway to enhance lipophagy activity, thereby reducing hepatic inflammation and apoptotic damage induced by high-fat diets [32]. Ursolic acid ameliorates metabolic dysfunction-associated steatotic liver disease by inhibiting SPP1 protein activity and suppressing Th17 cell differentiation [33]. Collectively, the regulatory effects of both PFF and PFS on glucolipid metabolism are likely attributable to these flavonoid and saponin constituents.

### 3.2. PFF and PFS Reduced Lipid Accumulation in High-Fat L02 Cells

**Figure 2:**
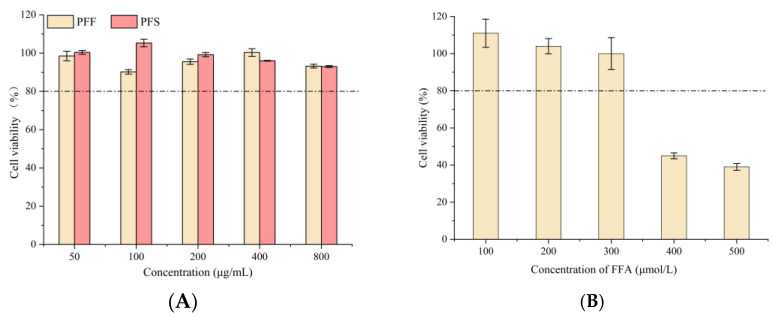

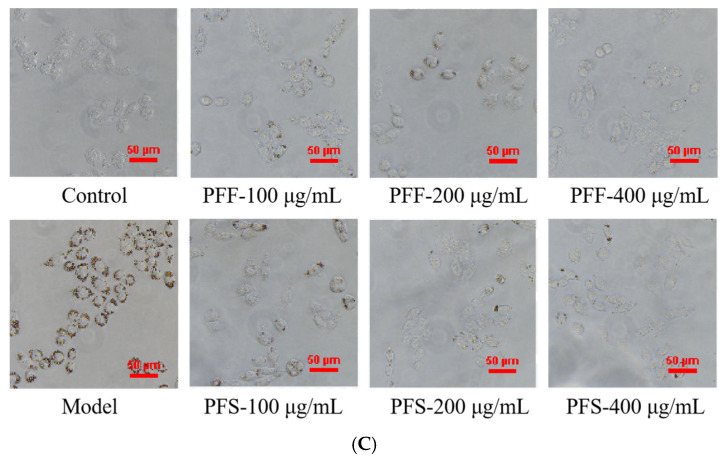
Comparison of PFF and PFS on cell viability of L02 cells (**A**) (The dotted line represents a cell viability of 80%); The effect of different concentrations of FFA on the survival rate of L02 cells (**B**); The effect of PFF and PFS on lipid accumulation (**C**). (n = 3).

As shown in Figure 2A, the survival rate of L02 cells at concentrations of PFF and PFS ranging from 50 to 800 µg/mL was over 80%, indicating that PFF and PFS were not toxic to L02 cells at these concentrations. Therefore, 100, 200, and 400 μg/mL of PFF and PFS were selected as the low, medium, and high concentration groups.

The excessive accumulation of FFA, a consequence of disordered metabolism closely linked to insulin resistance, type 2 diabetes mellitus, and obesity, not only exacerbates hepatic lipid droplet formation but also promotes hepatitis and fibrosis through the activation of inflammatory pathways [34]. The results of different concentrations of FFA acting on L02 cells were shown in Figure 2B. When the concentration of FFA was lower than 300 μmol/L, the cells could survive normally, and when it was higher than this concentration, cell death occurred. Therefore, 300 μmol/L FFA was chosen as the modeling concentration.

As shown in Figure 2C, compared with the control group, the lipid droplets aggregated at the edge of the cells in the model group were large and numerous, while the lipid droplets in the control group were almost invisible, indicating the success of modeling. At the concentrations of 100, 200 and 400 μg/mL, PFF and PFS significantly reduced the fat accumulation, and both samples had fewer lipid droplets in the cells with the increase in concentration, and the PFF group was almost the same as the control group at 400 μg/mL. With the same concentration of PFF and PFS, there were more lipid droplets in the PFS group than in the PFF group. Therefore, PFF and PFS could effectively reduce the fat accumulation in high-fat L02 cells, and the effect of PFF was better than that of PFS.

### 3.3. Effects of PFF and PFS on TG, TCHO, and LDL in High-Fat L02 Cells

**Figure 3:**
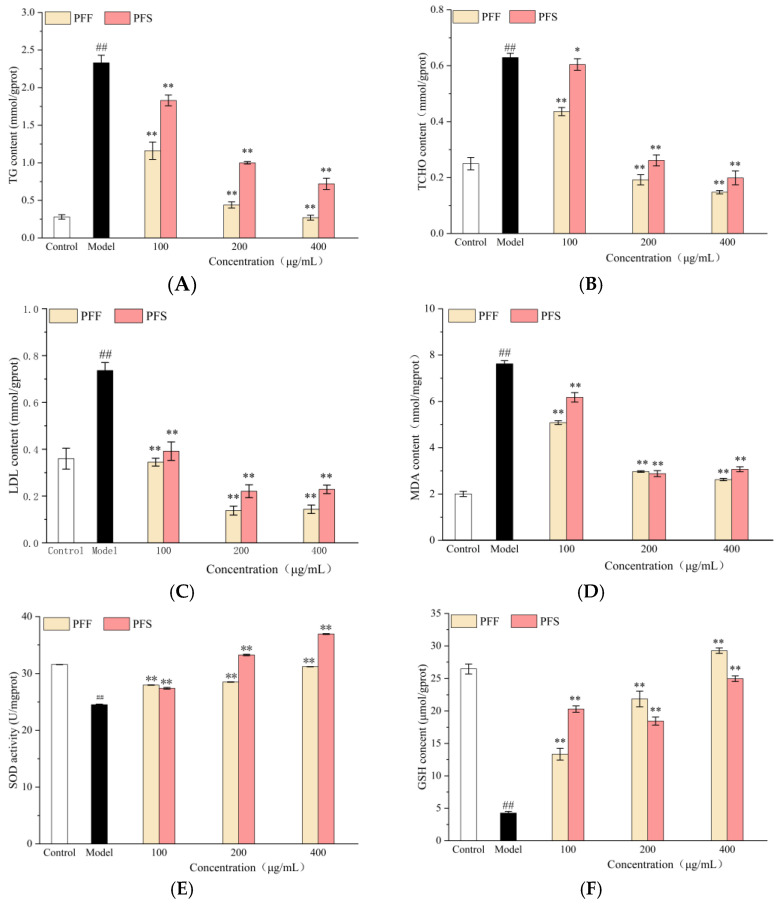
Effects of PFF and PFS on TG (**A**), TCHO (**B**), LDL (**C**), MDA (**D**), SOD (**E**) and GSH content (**F**) in high-fat L02 cells. (* *p* < 0.05 and ** *p* < 0.01, compared to the model group; ^##^ *p* < 0.01, compared to the control group) (n = 3).

In Figure 3A, the intracellular TG content decreased with the increase in sample concentration, and the effect of PFF was significantly better than that of PFS at the same concentration, and the cellular TG content in the sample group tended to be higher than that in the normal group when PFF was 400 μg/mL.

In Figure 3B, the intracellular TCHO content decreased with the increase in sample concentration, and at the same concentration, the effect of PFF was significantly better than that of PFS, and at the low concentration, the effect of PFS on intracellular TCHO content was not as significant as that of PFF, but there was a tendency to decrease (*p* < 0.05). When the concentration of PFF was greater than 100 μg/mL, the intracellular cholesterol content was lower than that of the normal group.

In Figure 3C, the intracellular LDL content of the sample groups was significantly lower than that of the model group at concentrations of 100–400 μg/mL (*p* < 0.01), and the effect of PFS on LDL reduction was not as effective as that of PFF. When the concentration exceeded 100 μg/mL, the two sample groups were able to lower the intracellular LDL content below that of the normal group.

These results corroborate our earlier observations of reduced lipid accumulation, as demonstrated by oil Red O staining, providing quantitative validation of the lipid-lowering efficacy of both PFF and PFS. Flavonoids are recognized for their capacity to inhibit pancreatic lipase activity and modulate lipid metabolism, thereby attenuating adipogenesis, while lipids facilitate the solubilization and transport of lipophilic flavonoids by forming mixed micelles, thereby enhancing their bioavailability [35]. The superior lipid-reducing performance of PFF suggests that it may be either a higher concentration of specific bioactive constituents or enhanced bioavailability, enabling more effective suppression of lipogenesis and promotion of lipolytic catabolism.

As can be seen from Figure 3D, the MDA content was significantly increased in the model group compared to the normal group, and the MDA content was significantly decreased in the sample group compared to the model group. As the concentration of PFF and PFS increased, the MDA content decreased.

The effect of the samples on intracellular SOD activity was shown in Figure 3E. The intracellular SOD activity in the model group was significantly decreased, and after the addition of the samples, there was a significant increase in the SOD activity, and the SOD activity increased with the increase in the sample concentration. When the concentration was 200 and 400 μg/mL, the SOD activity of the PFS group was higher than that of the PFF group, indicating that PFF was not as effective as PFS in enhancing the intracellular SOD activity at medium and high concentrations.

From Figure 3F, the GSH content in the model group decreased significantly, indicating that FFA could significantly inhibit the production of cellular GSH. The GSH content in high-fat L02 cells was elevated to different degrees in all sample groups compared to the control. The intracellular GSH content increased with the increase in PFF concentration, but there was no significant concentration dependence on PFS. At low concentrations, PFS was more effective than PFF in increasing GSH content, but at middle and high concentrations, PFF was more effective than PFS.

Both PFF and PFS treatments effectively reduced MDA levels and increased the activities of SOD and GSH, which not only confirm their antioxidant activities but also establish a link between their lipid-lowering effects and antioxidant mechanisms. We speculate that the lipid-lowering efficacy may be partly attributed to the alleviation of oxidative stress through free radical scavenging, thereby improving lipid peroxidation.

### 3.4. PFF and PFS Ameliorated Mitochondrial Membrane Potential Decrease in High-Fat L02 Cells

**Figure 4:**
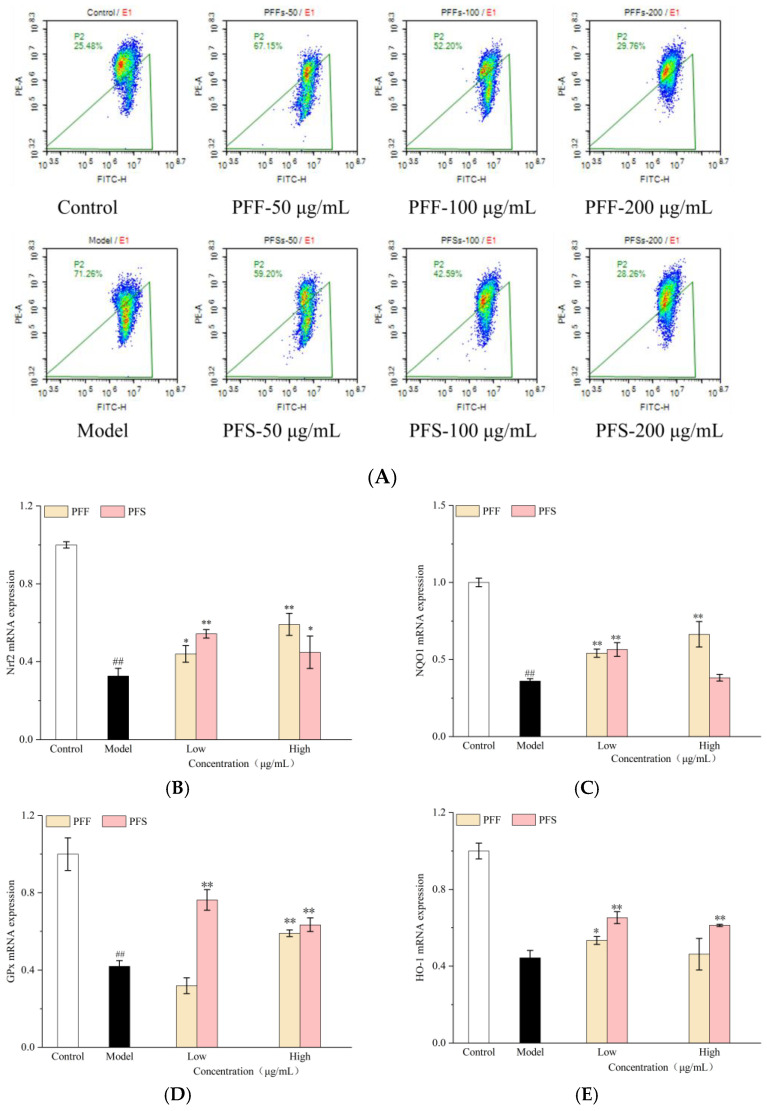

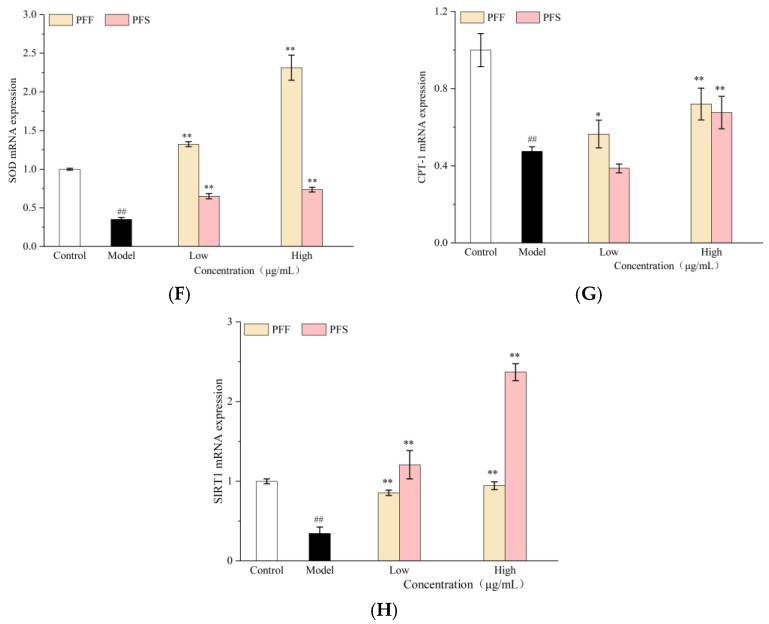
Effects of PFF and PFS on the mitochondrial membrane potential in high-fat L02 cells (**A**). Effects of PFF and PFS on mRNA expression of genes related to Nrf2/ARE pathway. (**B**): Nrf2; (**C**): NQO1; (**D**): GPx; (**E**): HO-1; (**F**): SOD; (**G**): CPT-1; (**H**): SIRT1 (n = 3). (*) *p* < 0.05 and (**) *p* < 0.01 compared to model group, (##) *p* < 0.01 compared to control group.

Mitochondria are essential organelles that supply energy for cellular activities, and the mitochondrial membrane potential serves as the driving force for ATP production [36]. A decline in mitochondrial membrane potential, often observed during the early stages of apoptosis, not only affects cellular energy metabolism but also affects the regulation of inflammatory and immune responses [37]. As shown in Figure 4A, the cells in the model group showed a decrease in mitochondrial membrane potential, which increased by 45.78% compared with the cells in the control group, indicating that FFA exerted a significant impact on the mitochondrial membrane potential. When PFF and PFS were applied to high-fat L02 cells, the proportion of cells with decreased mitochondrial membrane potential was significantly reduced compared with that of the model group, indicating that PFF and PFS were effective in ameliorating the decrease in mitochondrial membrane potential. The effects of the samples on the cells showed a significant concentration dependence on the sample concentration. At a sample concentration of 200 μg/mL, the proportion of cells exhibiting reduced mitochondrial membrane potential was 29.76% in the PFF-treated group and 28.26% in the PFS-treated group, closely approaching the level observed in the control group. The effect of PFS on mitochondrial membrane potential was greater than that of PFF at the same concentration.

### 3.5. Effects of PFF and PFS on the Expression of Nrf2/ARE Pathway-Related Genes

This study further investigates the mechanisms of action of PFF and PFS at the level of gene expression. Nrf2 is a cellular adaptive antioxidant master controller and an important mediator of glucose, lipid and energy metabolism in adipose tissue [38]. As shown in Figure 4B, the relative expression of Nrf2 mRNA in the model group was significantly lower than that in the control group. The action of PFF on the L02 cells could elevate the expression of the gene in a concentration-dependent manner, with the effect being more pronounced at higher concentrations. PFS could also enhance gene expression, but the effect was more pronounced at lower concentrations. This observation is consistent with the previously enhanced oxidative stress status (elevated MDA, reduced SOD and GSH) and mitochondrial dysfunction (decreased mitochondrial membrane potential).

The NQO1 gene could encode an intracellular protective reductase, which is capable of protecting the cells from oxidative damage [39]. As could be seen from Figure 4C, the relative expression of NQO1 mRNA in the model group was significantly reduced compared with that in the control group, but the mRNA expression was increased by the effects of PFF and PFS. The relative expression of NQO1 mRNA in the cells at the high concentration of PFF was higher than that at the low concentration, but there was no concentration dependence of NQO1 mRNA expression on PFS.

GPx, as an important peroxidation-degrading enzyme, is one of the important indicators of antioxidant effects [40]. As shown in Figure 4D, the relative expression of GPx mRNA in the model group was significantly lower than that in the control group, and the high concentration of PFS acting on the cells could significantly increase the expression of GPx mRNA in L02 cells, and both high and low concentrations of PFF could significantly increase the expression of this gene.

HO-1 can effectively ameliorate metabolic diseases through the lipocalin-dependent pathway [41]. As shown in Figure 4E, by analyzing the HO-1 mRNA expression, the model group showed a significant decrease in the expression of this gene, and PFS significantly increased the expression of this gene, but the effect of PFF was not obvious.

SOD could effectively destroy reactive oxygen species. As could be seen from Figure 4F, the relative expression of SOD mRNA was significantly increased by the effects of PFF and PFS, and the expression of this gene in the cells after the effect of PFF could be significantly higher than that of the control group by 2 times.

The upregulation of Nrf2 promoted the expression of cytoprotective genes (NQO1, HO-1) regulated by downstream antioxidant response elements (ARE), as well as key antioxidant genes (GPx, SOD). This coordinated activation strongly suggests that the antioxidant effects of PFF and PFS are not limited to isolated molecular targets, but rather involve the activation of the upstream Nrf2 signaling pathway, thereby enhancing the cellular antioxidant defense network in a coordinated manner [42,43].

Some studies have reported a correlation between silent information regulator 1 (SIRT1) and human obesity. SIRT1, as an energy-sensing molecule, controls transcriptional regulation of cellular energy status, deacetylates many transcription factors and histones involved in energy regulation, and promotes fat mobilization in white adipocytes by inhibiting PPARγ [44,45]. Lipid consumption starts with the activation of fatty acids to generate lipoyl CoA, which then enters the mitochondria for fatty acid β-oxidation, and carnitine palmitoyltransferase-1 (CPT-1) is a key rate-limiting enzyme in mitochondrial fatty acid oxidation and plays a very important role [46].

As shown in Figure 4G and Figure 4H, the relative mRNA expression levels of CPT-1 and SIRT1 decreased to 0.47 and 0.34, respectively, in the model group relative to the control group, suggesting that FFA inhibited lipid metabolism in L02 cells. The relative mRNA expression of CPT-1 and SIRT1 was significantly increased by PFF and PFS samples, and the activation of these two genes was concentration-dependent, in which the relative mRNA expression of SIRT1 was higher than that of the normal group, and the high concentration reached 0.34. The relative mRNA expression of SIRT1 was higher than that of the control group. The relative expression of SIRT1 mRNA in the cells under the effect of PFS was higher than that in the control group, and the high concentration reached 2.36, indicating that PFF and PFS could inhibit the accumulation of fat by improving lipid metabolism. In summary, PFF and PFS can improve fat metabolism by regulating the expression of Nrf2/ARE pathway-related genes.

Studies have demonstrated that SIRT1 can prevent the ubiquitination and degradation of Nrf2 through deacetylation, facilitating its translocation into the nucleus and thereby activating the transcription of downstream antioxidant genes [47]. The observed upregulation of SIRT1 is consistent with the previously activated Nrf2-mediated antioxidant pathway following treatment with PFF and PFS. Therefore, it is reasonable to hypothesize that PFF and PFS may enhance Nrf2-mediated antioxidant responses by activating SIRT1, thereby alleviating mitochondrial damage caused by lipid peroxidation and providing a functionally competent environment for β-oxidation. Concurrently, activated SIRT1 and upregulated CPT-1 work synergistically to drive fatty acid β-oxidation. This creates a beneficial cycle of coordinated antioxidant and lipid metabolism effects, collectively ameliorating high-fat diet-induced hepatocyte injury.

### 3.6. PFF and PFS Reduced Lipid Accumulation in C. elegans

**Figure 5:**
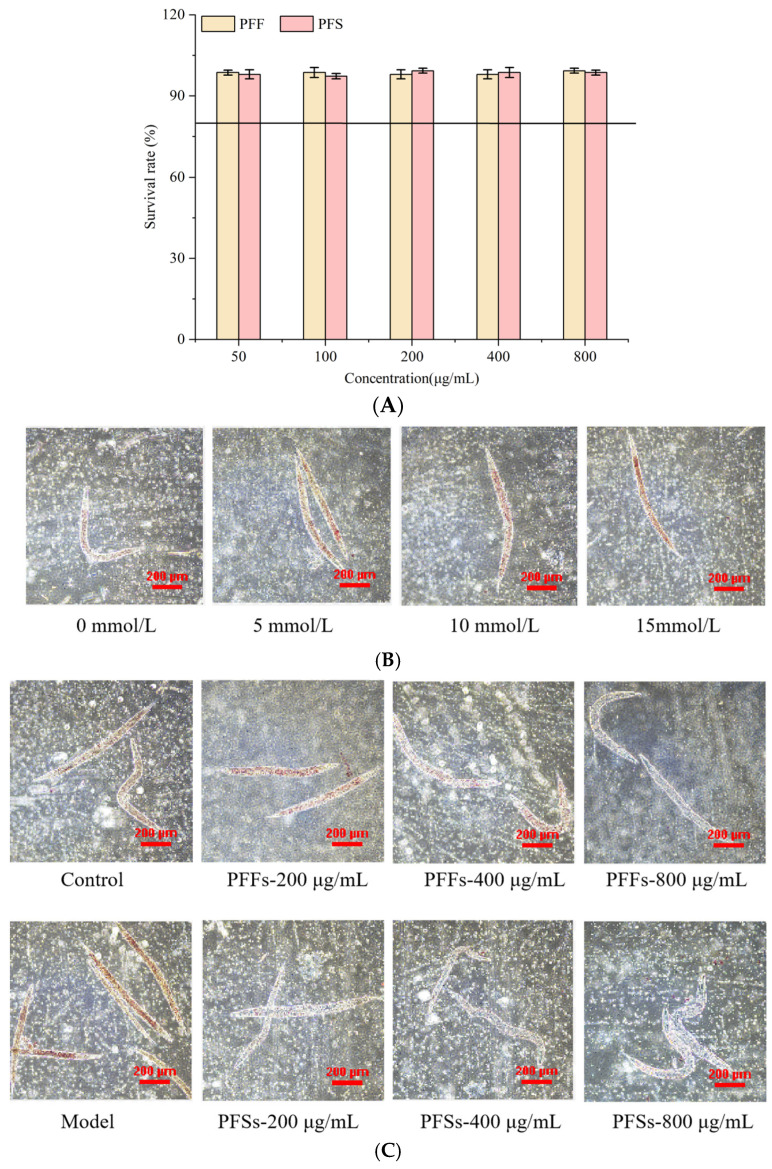
Determination of toxicity of PFF and PFS to *C. elegans* (**A**) (The solid line in (**A**) means the survival rate of worms was 80%). Effect of different glucose concentrations on the fat content of *C. elegans* (**B**). Effect of PFF and PFS on fat content (**C**) (n = 3).

As shown in Figure 5A, the survival rate of worms was more than 80% when the concentrations of PFF and PFS were in the range of 50–800 μg/mL, so 800 μg/mL was chosen as the maximum concentration of PFF and PFS. It has been shown that high-fat diets can induce obesity, so nematodes were provided with high-fat diets to promote fat accumulation [48]. As shown in Figure 5B, the colored area in worms that ingested glucose was significantly larger than that of worms that did not ingest glucose, suggesting that glucose ingestion promotes fat accumulation in worms. Different concentrations of glucose had different effects on the fat accumulation of worms. When the concentration of glucose in the NGM medium was 5–15 mmol/L, the red area of the worms was larger, indicating that the fat accumulation was greater. Therefore, 15 mmol/L glucose was chosen as the modeling concentration.

The results of determining the effects of PFF and PFS on fat accumulation of *C. elegans* by oil red O staining are shown in Figure 5C. The red-covered area of worms in the model group was significantly higher than that in the control group, indicating successful modeling. After feeding PFF and PFS to the worms, the fat content was reduced to different degrees. This finding further corroborates the conclusions drawn from our L02 cell model, collectively confirming the lipid-lowering effects of both interventions. As the concentration of PFF increased, the lipid deposition of worms decreased significantly. Under the effect of PFF at 400 μg/mL, the degree of lipid accumulation was basically the same as that of the control group, and under the action of 800 μg/mL of PFF, the amount of fat accumulation was significantly lower than that of the control group. The lipid content of worms under the effect of PFS was significantly lower than that of the model and control groups. At the same concentration, the lipid-lowering effect of PFS on *C. elegans* was significantly better than that of PFF.

### 3.7. Effects of PFF and PFS on TG, TCHO, LDL, CAT and MDA in C. elegans

**Figure 6:**
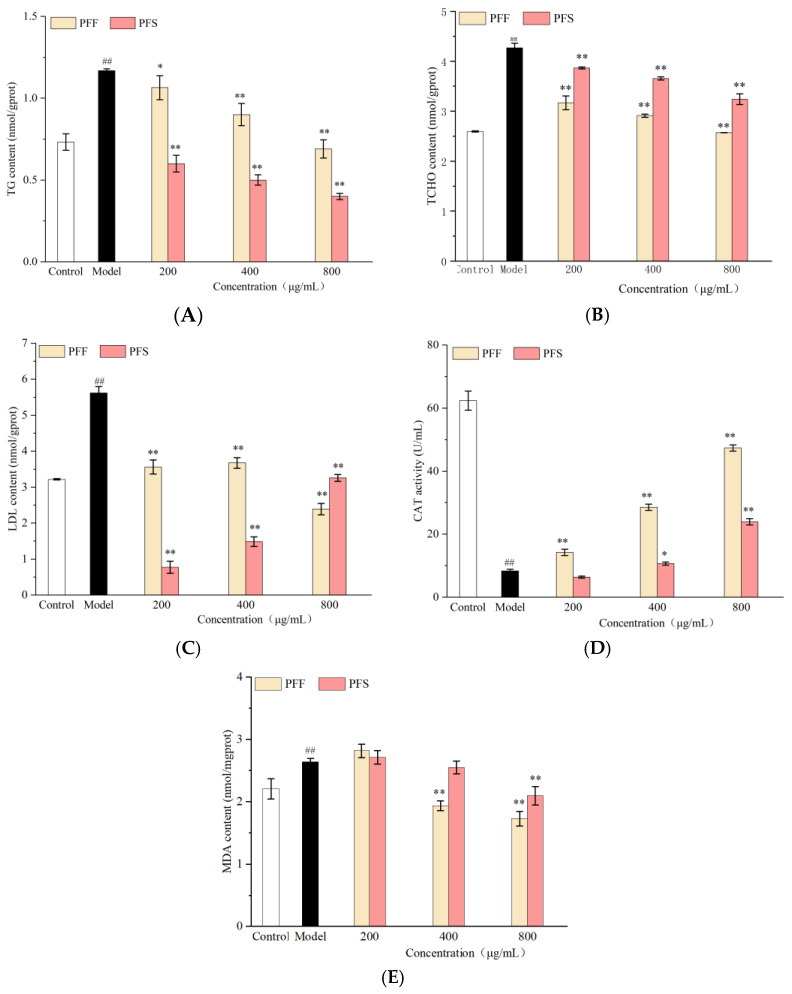

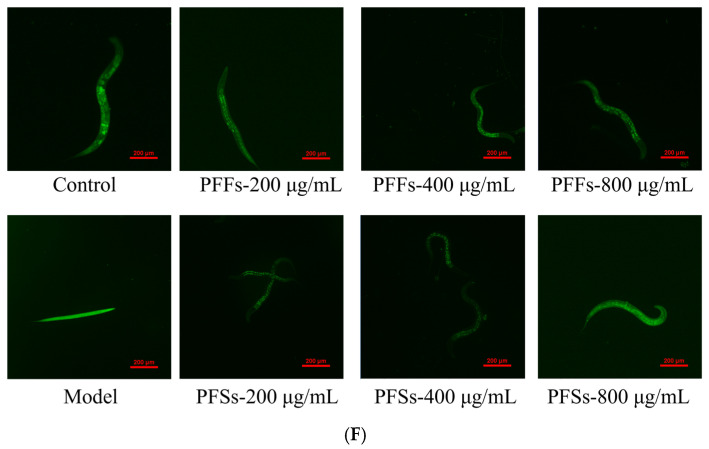
Effects of PFF and PFS on TG (**A**), TCHO (**B**), LDL (**C**), CAT (**D**), MDA (**E**) and ROS content (**F**) in *C. elegans*. (* *p* < 0.05 and ** *p* < 0.01, compared to the model group; ^##^ *p* < 0.01, compared to the control group) (n = 3).

As shown in Figure 6A–C, the TG, TCHO, and LDL content of the model group were significantly higher than those of the control group (*p* < 0.01), indicating that the high-fat diet promoted the accumulation of TCHO and TG and increased the content of LDL in worms. After feeding PFF and PFS to the worms, all three indicators were reduced.

As shown in Figure 6A, when the PFF administration was 200–400 μg/mL, the TG content was lower than that of the model group. After the concentration increased to 800 μg/mL, it reduced the TG content to the normal level. At PFS concentrations of 200–800 μg/mL, TG levels were consistently lower than those in the control group. At the same concentration, the ability of PFS to regulate the TG of worms was stronger than that of PFF, which was consistent with the results of oil red O staining.

As shown in Figure 6B, the TCHO content of worms fed with PFF and PFS remained higher than that of the control group; it was significantly lower than that of the model group (*p* < 0.01). The effect of the samples on TCHO at the same concentration was superior for PFF than PFS. This was inconsistent with the effect of the samples on TG and may be related to the different lipid-lowering mechanisms of PFF and PFS.

As can be seen from Figure 6C, PFF and PFS significantly reduced the LDL content of worms. Under the effect of low concentration in PFF, the LDL content was significantly lower than model group (*p* < 0.01). Under the effect of high concentration of PFF, the LDL content was lower than control group. The effect of PFS on LDL was better than that of PFF at 200–400 μg/mL, but when the concentration was 800 μg/mL, the effect of PFF was better than that of PFS. It could be concluded that PFF and PFS could significantly reduce the lipid content of worms, but the effects on different lipid indexes were different.

As shown in Figure 6D, the CAT activity of the model group was significantly lower than that of the control group, indicating that high sugar stimulation could lead to oxidative damage in worms. The effects of PFF on worms at the concentrations of 200–800 μg/mL increased the CAT activity in relation to the model group, but the PFS only had an effect on the CAT activity at high concentrations.

From Figure 6E, it could be seen that the MDA content of the model group was significantly higher than that of the control group, and the medium and high concentrations of PFF could inhibit the MDA production of the high-fat worms, and only high concentrations of PFS inhibited MDA production. At the same concentration, the inhibitory effect of PFF on MDA production was better than that of PFS.

Taken together, these analyses confirm that both PFF and PFS effectively reduce lipid content in C. elegans. However, their effects on specific lipid indicators differ, suggesting distinct and potentially complementary mechanisms of action.

### 3.8. Effects of PFF and PFS on ROS in C. elegans

Recent studies have shown that ROS levels in adipose tissue are closely related to obesity and metabolic diseases, and that excess reactive oxygen species cause lipid peroxidation, oxidative damage to nucleic acids, and oxidation of monosaccharides, which can lead to diseases such as obesity and metabolic diseases [49].

As shown in Figure 6F, the fluorescence intensity of the model group was significantly higher than control group, indicating that there was a certain link between obesity and the production of ROS. After different concentrations of PFF and PFS were applied to worms, the fluorescence intensity was significantly lower than that of the model group. There was no significant difference in the fluorescence intensity of PFF at 200, 400, and 800 μg/mL when PFF was applied to worms. However, as the concentration of PFS increased, the fluorescence intensity of the worms decreased and was even lower than that of the control group, which indicated that PFS was more effective in inhibiting the production of ROS than PFF.

## 4. Conclusions

This study successfully extracted flavonoids (PFF) and saponins (PFS) compounds from *P. fortuneana* fruits, a dual-use medicinal and edible plant. It systematically evaluated their lipid-modulating activities and underlying mechanisms in high-fat-induced L02 hepatocytes and *C. elegans* models. The results demonstrate that PFF and PFS not only significantly mitigate intracellular lipid accumulation and nematode fat deposition but also enhance antioxidant capacity and promote lipid catabolism through the activation of the Nrf2/ARE pathway and the upregulation of key lipid-oxidizing genes, including CPT-1 and SIRT1. Given the established roles of SIRT1 and Nrf2 in glycolipid metabolism, these bioactive components derived from *P. fortuneana* may possess certain regulatory functions within the interconnected network of glycolipid metabolism.

Future research should focus on validating the anti-obesity effects and determining appropriate dosage levels in mammalian models, while also addressing critical aspects such as stability within food matrices, bioavailability, and formulation compatibility. Such investigations would facilitate the translation of *P. fortuneana* bioactive compounds into practical applications in the health food sector, thereby contributing to scientifically grounded dietary strategies for obesity intervention.

## Figures and Tables

**Table 1:** The primer sequences of related genes in L02 cells.

Genes	Primer Sequences
*Nrf2*	Forward: *CGGTATGCAACAGGACATTG*
	Reverse: *ACTGGTTGGGGTCTTCTGTG*
*HO-1*	Forward: *CCAGGCAGAGAATGCTGAGT*
	Reverse: *GTAGACAGGGGCGAAGACTG*
*NQO1*	Forward: *CTGATCGTACTGGCTCACTC*
	Reverse: *GAACAGACTCGGCAGATAC*
*SOD*	Forward: *GAAGGTGTGGGGAAGCATTA*
	Reverse: *ACCACAAGCCAAACGACTTC*
*CPT-1*	Forward: *CCTCCGTAGCTGACTCGGTA*
	Reverse: *GGAGTGACCGTGAACTGAAAG*
*SIRT1*	Forward: *GCCTCATCTGCATTTTGATG*
	Reverse: *TCTGGCATGTCCCACTATCA*
*GPx*	Forward: *GTCAATGTTGCATCACAATGTGG*
	Reverse: *CAGCTTCTTCACGTCCTTCTCAAT*
*β-actin*	Forward: *CTTAGTTGCGTTACACCCTTTC*
	Reverse: *ACCTTCACCGTTCCAGTTTT*

**Table 2:** LC-MS/MS analysis results of flavonoids and saponins in PFF and PFS.

Nr	RT/min	Molecule Formula	Molecular Weight	Fragment Ions	Ionization Mode	Compound
PFF						
1	8.16	C_15_H_14_O_6_	290.0793	291.0865	[M+H]^+^	Epicatechin
2	11.9	C_21_H_20_O_12_	464.376	464.1000	[M+H]^+^	Isoquercetin
3	12.43	C_15_H_10_O_6_	287.0538	286.0471	[M+H]^+^	Rutin
4	12.91	C_27_H_32_O_16_	612.1690	613.2	[M+H]^+^	Hydroxysafflor yellow A
5	13.11	C_20_H_18_O_5_	338.1154	339.1	[M+H]^+^	5,7,4′Trihydroxy-6-iso Pentenyl isoflavone
6	8.16	C_15_H_10_O_7_	302.0427	300.9	[M-H]^−^	Quercetin
7	9.35	C_15_H_10_O_5_	270.0528	269	[M-H]^−^	Baicalein
8	11.9	C_21_H_20_O_12_	464.09627	463.0889	[M-H]^−^	Myricitrin
PFS						
1	11.9	C_30_H_50_O	426.386165	427.4	[M+H]^+^	Shionone
2	12.48	C_30_H_48_O_4_	472.35526	473.4	[M+H]^+^	Crategolic acid
3	12.93	C_30_H_48_O_3_	456.360345	455.3532	[M+H]^+^	Ursolic Acid
4	16.47	C_18_H_32_O_5_	328.22536	327.2180	[M-H]^−^	Cocoa butter acid F
5	17.26	C_18_H_34_O_5_	330.2411	329.2338	[M-H]^−^	(15z)-9,12, 13-Trihydroxy-15-octadecenoic acid

## Data Availability

The data that support the findings of this study are available from the corresponding author upon reasonable request.

## References

[1] Gomez-Delgado F., Katsiki N., Lopez-Miranda J., Perez-Martinez P. (2020). Dietary habits, lipoprotein metabolism and cardiovascular disease: From individual foods to dietary patterns. Crit. Rev. Food Sci. Nutr..

[2] Plutzky J. (2000). Emerging concepts In metabolic abnormalities associated with coronary artery disease. Curr. Opin. Cardiol..

[3] Rashid S., Genest J. (2007). Effect of obesity on high-density lipoprotein metabolism. Obesity.

[4] Deng X.J., Hou Y., Zhou H.J., Li Y.L., Xue Z.Q., Xue X.T., Huang G.H., Huang K.L., He X.Y., Xu W.T. (2021). Hypolipidemic, anti-inflammatory, and anti-atherosclerotic effects of tea before and after microbial fermentation. Food Sci. Nutr..

[5] Mazza A., Nicoletti M., Lenti S., Torin G., Rigatelli G., Pellizzato M., Fratter A. (2021). Effectiveness and Safety of Novel Nutraceutical Formulation Added to Ezetimibe in Statin-Intolerant Hypercholesterolemic Subjects with Moderate-to-High Cardiovascular Risk. J. Med. Food.

[6] Guo W.L., Pan Y.Y., Li L., Li T.T., Liu B., Lv X.C. (2018). Ethanol extract of *Ganoderma lucidum* ameliorates lipid metabolic disorders and modulates the gut microbiota composition in high-fat diet fed rats. Food Funct..

[7] Singh S.P., Sashidhara K.V. (2017). Lipid lowering agents of natural origin: An account of some promising chemotypes. Eur. J. Med. Chem..

[8] Wang L., Li R., Zhang Q., Liu J., Tao T., Zhang T., Wu C., Ren Q., Pu X., Peng W. (2022). *Pyracantha fortuneana* (Maxim.) Li: A comprehensive review of its phytochemistry, pharmacological properties, and product development. Front. Sustain. Food Syst..

[9] Li H., Fang W., Wang Z., Chen Y. (2022). Physicochemical, biological properties, and flavour profile of *Rosa roxburghii* Tratt, *Pyracantha fortuneana*, and Rosa laevigata Michx fruits: A comprehensive review. Food Chem..

[10] Yuan C.F., Wang C.D., Bu Y.Q., Xiang T.X., Huang X.N., Wang Z.W., Yi F.P., Ren G.S., Liu G.L., Song F.Z. (2010). Antioxidative and immunoprotective effects of *Pyracantha fortuneana* (Maxim.) Li polysaccharides in mice. Immunol. Lett..

[11] Yuan C.F., Li Z.H., Yi M.H., Wang X.X., Peng F., Xiao F.X., Chen T., Wang C.D., Mushtaq G., Kamal M.A. (2015). Effects of Polysaccharides from Selenium-enriched *Pyracantha fortuneana* on Mice Liver Injury. Med. Chem..

[12] Wang H., Ye Y.H., Wang H.H., Liu J., Liu Y.J., Jiang B.W. (2019). HPLC-QTOF-MS/MS profiling, antioxidant, and α-glucosidase inhibitory activities of *Pyracantha fortuneana* fruit extracts. J. Food Biochem..

[13] Rubin J.K., Hinrichs-Krapels S., Hesketh R., Martin A., Herman W.H., Rubino F. (2016). Identifying Barriers to Appropriate Use of Metabolic/Bariatric Surgery for Type 2 Diabetes Treatment: Policy Lab Results. Diabetes Care.

[14] Li Y., Mei M., Wang Q., Gen L., Hao K., Zhong R., Mo T., Jiang J., Zhu W. (2024). Structural characteristics and anti-photoaging effect of *Pyracantha fortuneana* fruit polysaccharides in vitro and in vivo. Int. J. Biol. Macromol..

[15] Wang Y., Shao Q., Yang X., Su K., Li Z., Yang Y., Yuan X., Chen R. (2024). Diversity in *Pyracantha fortuneana* fruits maturity stages enables discrepancy in the phenolic compounds, antioxidant activity, and tyrosinase inhibitory activity. J. Food Sci..

[16] Yin Y., Huang Y., Yang W., Yuan J., Xie M., Miao Y., Yu J., Wang J., Zhang X., Wang B. (2023). A novel flavonoid and other constituents from *Rubus rosifolius* S.Vidal (Rosaceae). Nat. Prod. Res..

[17] Li G., Yu S., Zhou Y.H., Chen Q.F. (2013). Spectrophotometric Determination of Flavonoids Content in Leaves of *Fagopyrum cymosum* Complex. Asian J. Chem..

[18] Hu T., Guo Y.Y., Zhou Q.F., Zhong X.K., Zhu L., Piao J.H., Chen J., Jiang J.G. (2012). Optimization of Ultrasonic-Assisted Extraction of Total Saponins from Eclipta prostrasta L. Using Response Surface Methodology. J. Food Sci..

[19] Xu X.Y., Hu J.P., Wu M.M., Wang L.S., Fang N.Y. (2015). CCAAT/enhancer-binding protein CEBP-2 controls fat consumption and fatty acid desaturation in *Caenorhabditis elegans*. Biochem. Biophys. Res. Commun..

[20] Xu Y., Ke H.H., Li Y.T., Xie L.H., Su H.M., Xie J.H., Mo J.L., Chen W. (2021). Malvidin-3-*O*-Glucoside from Blueberry Ameliorates Nonalcoholic Fatty Liver Disease by Regulating Transcription Factor EB-Mediated Lysosomal Function and Activating the Nrf2/ARE Signaling Pathway. J. Agric. Food Chem..

[21] Abdulkhaleq L.A., Assi M.A., Noor M.H.M., Abdullah R., Saad M.Z., Taufiq-Yap Y.H. (2017). Therapeutic uses of epicatechin in diabetes and cancer. Vet. World.

[22] Zhang C., Shi Z., Lei H., Wu F., Chen C., Cao Z., Song Y., Zhang C., Zhou J., Lu Y. (2023). Dietary Isoquercetin Reduces Hepatic Cholesterol and Triglyceride in NAFLD Mice by Modulating Bile Acid Metabolism via Intestinal FXR-FGF15 Signaling. J. Agric. Food Chem..

[23] Liu Y., Sun Z., Dong R., Liu P., Zhang X., Li Y., Lai X., Cheong H.-F., Wu Y., Wang Y. (2024). Rutin ameliorated lipid metabolism dysfunction of diabetic NAFLD via AMPK/SREBP1 pathway. Phytomedicine.

[24] Li F., Zhang L., Zhang X., Fang Q., Xu Y., Wang H. (2024). Rutin alleviates Pb-induced oxidative stress, inflammation and cell death via activating Nrf2/ARE system in SH-SY5Y cells. NeuroToxicology.

[25] Feng X., Du M., Li S., Zhang Y., Ding J., Wang J., Wang Y., Liu P. (2023). Hydroxysafflor yellow A regulates lymphangiogenesis and inflammation via the inhibition of PI3K on regulating AKT/mTOR and NF-κB pathway in macrophages to reduce atherosclerosis in ApoE-/- mice. Phytomedicine.

[26] Wang W., Liu M., Fu X., Qi M., Zhu F., Fan F., Wang Y., Zhang K., Chu S. (2024). Hydroxysafflor yellow A ameliorates alcohol-induced liver injury through PI3K/Akt and STAT3/NF-κB signaling pathways. Phytomedicine.

[27] Zhu X., Ding G., Ren S., Xi J., Liu K. (2024). The bioavailability, absorption, metabolism, and regulation of glucolipid metabolism disorders by quercetin and its important glycosides: A review. Food Chem..

[28] Liu N., Cui X., Guo T., Wei X., Sun Y., Liu J., Zhang Y., Ma W., Yan W., Chen L. (2024). Baicalein Ameliorates Insulin Resistance of HFD/STZ Mice Through Activating PI3K/AKT Signal Pathway of Liver and Skeletal Muscle in a GLP-1R-Dependent Manner. Antioxidants.

[29] Gurumayum S., Basumatary D., Sarma P., Saikia K., Swargiary D., Akhtar S.A., Saikia A., Borah J.C. (2024). Dietary vegetable *Sarcochlamys pulcherrima* Gaud. And its bioactive compound myricitrin promotes white adipose browning in obese models via AMPK/SIRT1/UCP1 upregulation. Food Biosci..

[30] Takahashi H., Morimoto H., Tanaka M., Inoue H., Goto T., Kawada T., Uehara M., Takahashi N. (2024). Myricetin and myricitrin indirectly and directly increases uncoupling protein-1 mRNA expression in C3H10T1/2 beige adipocytes. Biochem. Biophys. Res. Commun..

[31] Jaiswal V., Lee H.-J. (2023). Pharmacological Properties of Shionone: Potential Anti-Inflammatory Phytochemical against Different Diseases. Molecules.

[32] Li T., Wang H., Dong S., Liang M., Ma J., Jiang X., Yu W. (2022). Protective effects of maslinic acid on high fat diet-induced liver injury in mice. Life Sci..

[33] Zheng Y., Zhao L., Xiong Z., Huang C., Yong Q., Fang D., Fu Y., Gu S., Chen C., Li J. (2024). Ursolic acid targets secreted phosphoprotein 1 to regulate Th17 cells against metabolic dysfunction-associated steatotic liver disease. Clin. Mol. Hepatol..

[34] Qin W., Ding Y., Zhang W., Sun L., Weng J., Zheng X., Luo S. (2025). Small molecule-driven LKB1 deacetylation is responsible for the inhibition of hepatic lipid response in NAFLD. J. Lipid Res..

[35] Zhang J., Wang H., Ai C., Lu R., Chen L., Xiao J., Teng H. (2023). Food matrix-flavonoid interactions and their effect on bioavailability. Crit. Rev. Food Sci. Nutr..

[36] LeFort K.R., Rungratanawanich W., Song B.-J. (2024). Contributing roles of mitochondrial dysfunction and hepatocyte apoptosis in liver diseases through oxidative stress, post-translational modifications, inflammation, and intestinal barrier dysfunction. Cell. Mol. Life Sci..

[37] Popgeorgiev N., Gil C., Berthenet K., Bertolin G., Ichim G. (2024). Shedding light on mitochondrial outer-membrane permeabilization and membrane potential: State of the art methods and biosensors. Semin. Cell Dev. Biol..

[38] Annie-Mathew A.S., Prem-Santhosh S., Jayasuriya R., Ganesh G., Ramkumar K.M., Sarada D.V.L. (2021). The pivotal role of Nrf2 activators in adipocyte biology. Pharmacol. Res..

[39] Zhang L., Dasuri K., Fernandez-Kim S.O., Bruce-Keller A.J., Keller J.N. (2016). Adipose-specific ablation of *Nrf2* transiently delayed high-fat diet-induced obesity by altering glucose, lipid and energy metabolism of male mice. Am. J. Transl. Res..

[40] Cao L.F., Li L.S., Spruell C., Xiao L., Chakrabarti G., Bey E.A., Reinicke K.E., Srougi M.C., Moore Z., Dong Y. (2014). Tumor-Selective, Futile Redox Cycle-Induced Bystander Effects Elicited by NQO1 Bioactivatable Radiosensitizing Drugs in Triple-Negative Breast Cancers. Antioxid. Redox Signal..

[41] Asayama K., Nakane T., Dobashi K., Kodera K., Hayashibe H., Uchida N., Nakazawa S. (2001). Effect of obesity and troglitazone on expression of two glutathione peroxidases: Cellular and extracellular types in serum, kidney and adipose tissue. Free. Radic. Res..

[42] He F., Ru X., Wen T. (2020). NRF2, a Transcription Factor for Stress Response and Beyond. Int. J. Mol. Sci..

[43] Morgenstern C., Lastres-Becker I., Demirdöğen B.C., Costa V.M., Daiber A., Foresti R., Motterlini R., Kalyoncu S., Arioz B.I., Genc S. (2024). Biomarkers of NRF2 signalling: Current status and future challenges. Redox Biol..

[44] Mohseni R., Sadeghabadi Z.A., Goodarzi M.T., Teimouri M., Nourbakhsh M., Azar M.R. (2018). Evaluation of Mn-superoxide dismutase and catalase gene expression in childhood obesity: Its association with insulin resistance. J. Pediatr. Endocrinol. Metab..

[45] Clark S.J., Falchi M., Olsson B., Jacobson P., Cauchi S., Balkau B., Marre M., Lantieri O., Andersson J.C., Jernås M. (2012). Association of Sirtuin 1 (*SIRT1*) Gene SNPs and Transcript Expression Levels with Severe Obesity. Obesity.

[46] Picard F., Kurtev M., Chung N.J., Topark-Ngarm A., Senawong T., de Oliveira R.M., Leid M., McBurney M.W., Guarente L. (2004). Sirt1 promotes fat mobilization in white adipocytes by repressing PPAR-γ. Nature.

[47] Zhuang K., Jiang X., Liu R., Ye C., Wang Y., Wang Y., Quan S., Huang H. (2021). Formononetin Activates the Nrf2/ARE Signaling Pathway Via Sirt1 to Improve Diabetic Renal Fibrosis. Front. Pharmacol..

[48] Lin C.X., Lin Y.Z., Chen Y., Xu J.N., Li J., Cao Y., Su Z.X., Chen Y.J. (2019). Effects of *Momordica* saponin extract on alleviating fat accumulation in *Caenorhabditis elegans*. Food Funct..

[49] Yu J., Qiu J., Zhang Z., Cui X.D., Guo W.X., Sheng M.Z., Gao M.Y., Wang D.M., Xu L.Y., Ma X.R. (2023). Redox Biology in Adipose Tissue Physiology and Obesity. Adv. Biol..

